# The regulatory roles of microRNAs toward pathogenesis and treatments in Huntington's disease

**DOI:** 10.1186/s12929-021-00755-1

**Published:** 2021-08-19

**Authors:** Chih-Wei Tung, Pin-Yu Huang, Siew Chin Chan, Pei-Hsun Cheng, Shang-Hsun Yang

**Affiliations:** 1 Department of Physiology, College of Medicine, National Cheng Kung University, Tainan, 70101 Taiwan; 2 Institute of Basic Medical Sciences, National Cheng Kung University, Tainan, 70101 Taiwan

**Keywords:** Huntington’s disease (HD), Pathogenesis, microRNA (miRNA), Gene regulation, miRNA-based therapy

## Abstract

Huntington’s disease (HD) is one of neurodegenerative diseases, and is defined as a monogenetic disease due to the mutation of *Huntingtin* gene. This disease affects several cellular functions in neurons, and further influences motor and cognitive ability, leading to the suffering of devastating symptoms in HD patients. MicroRNA (miRNA) is a non-coding RNA, and is responsible for gene regulation at post-transcriptional levels in cells. Since one miRNA targets to several downstream genes, it may regulate different pathways simultaneously. As a result, it raises a potential therapy for different diseases using miRNAs, especially for inherited diseases. In this review, we will not only introduce the update information of HD and miRNA, but also discuss the development of potential miRNA-based therapy in HD. With the understanding toward the progression of miRNA studies in HD, we anticipate it may provide an insight to treat this devastating disease, even applying to other genetic diseases.

## Background

Huntington’s disease (HD) is one of inherited diseases, and leads to neurodegeneration in patients. Due to the progression of this disease, the patients suffer severe motor and cognitive deficits till death, and unfortunately there is no cure for this disease yet. As a result, numbers of studies have tried to demonstrate on molecular mechanisms of this disease, and anticipate to identify or develop novel strategies for therapy. Recently, microRNA (miRNA) is a raising candidate to apply in gene therapy due to critically regulatory roles of gene expression inside cells. Taking advantages from specific characteristics of miRNAs, researchers have been developing miRNA-based therapy for different inherited diseases, and hope to alleviate or cure these diseases, including neuronal diseases. In this article, we will review the molecular pathogenesis of HD, and show the recent development of therapy for this disease. In addition, we will also review the applications of miRNAs targeting on neuronal pathogenesis, and discuss the pons and cons of this potential miRNA-based strategy.

## Huntington’s disease

### General introduction

#### Genetic information

Huntington’s disease (HD) is an autosomal dominant disease, and the clinic description of this disease was first reported by the American physician, George Huntington, in 1872. This disease belongs to rare diseases, and the prevalence rate of HD in the US and Canada is approximately 6.52–13.7 patients per 100,000 population [1]. Additionally, HD is a monogenic disease, and the disease-causing gene, *Huntingtin *(*HTT*; also known as *IT15*), was first identified in 1993 [2]. This *HTT* gene is located in chromosome 4p16.3 region, and has 67 exons to be translated into an approximately 350 kDa HTT protein. Most importantly, there is a critical expansion of CAG trinucleotide repeats in the exon 1 region of *HTT* gene, and patients carrying mutant *HTT* (*mHTT*) with more than 36 CAG repeats will develop the symptoms of HD gradually. Due to that the abnormally expanded CAG trinucleotides could be translated into glutamines, HD is also considered as one of polyglutamine (ployQ) diseases [2, 3]. Commonly, the onset of HD is approximately at 40 years of age [4], and the cause of death usually is suicide due to unaffordable conditions of symptoms for patients [5]. Unfortunately, these is still no cure for this disease, and physicians only could treat this disease by alleviation of clinic symptoms.

#### Pathology

HTT is dominantly expressed in brains, and mHTT forms toxic gain-of function proteins to disrupt neuronal functions, leading to cell death and neurodegeneration. The most affected brain regions in HD patients include deeper layers of the cortex and striatum, and mHTT also additionally influences other brain regions, such as hippocampus, hypothalamus and cerebellum [3, 6]. Within different brain regions, medium spiny neurons in striatum, including caudate and putamen, are considered as the most susceptible cells affected by mHTT, resulting in neuronal death, striatum atrophy and enlarged lateral ventricles in HD patients [6, 7]. Due to deficits of striatum, HD also causes in the imbalance of glutamate and dopamine activity, further disrupting the circuitry in basal ganglia [8]. As to the cellular hallmarks of HD neuropathology, nuclear aggregates, intranuclear inclusions (also known as inclusion bodies) and neuropil aggregates are typical characteristics in postmortem brains of HD patients [6, 9], which are also often used as an indicator for the severity of HD.

#### Clinical symptoms

Due to disruption of neuronal cells in brains, HD patients display cognitive and motor dysfunctions. In HD patients, they suffer different illness, such as emotional problems, mental deterioration, fatigue, difficulty thinking, memory loss, impaired sleep or daytime sleepiness [3, 9, 10]. In addition, these patients also show chorea, dystonia, involuntary movements and difficulty walking, speaking and swallowing because of the impaired ability of motor control in the central nervous system (CNS) [3, 9, 11]. With the worse cognitive and motor functions, HD patients change behaviors and gradually lose independence, leading to huge burdens for their families and societies.

### Molecular pathogenesis

#### Protein misfolding

**Fig. 1:**
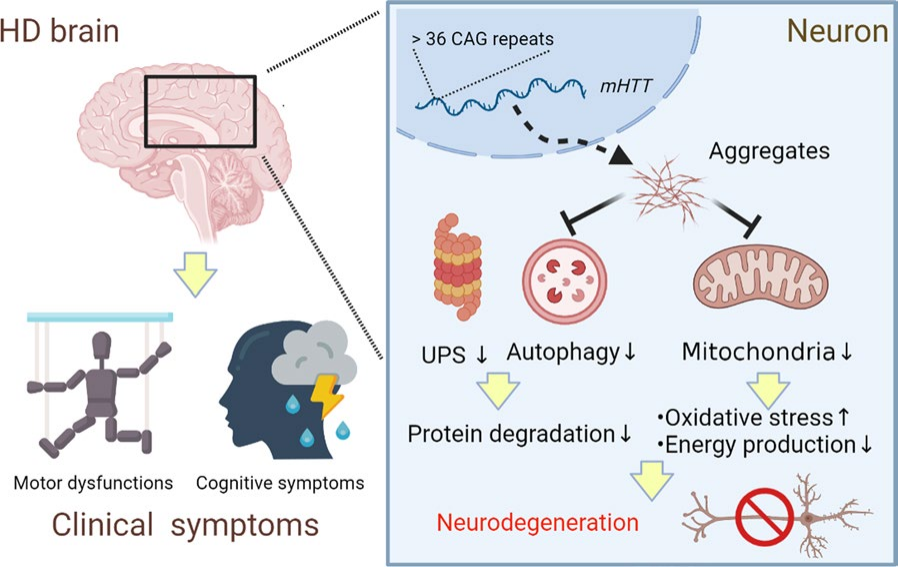
mHTT leads to cellular dysfunctions and clinical symptoms in HD patients. mHTT is transcribed from *mHTT* gene with more than 36 CAG repeats, and then forms aggregates to cause cellular dysfunctions, such as in protein degradation systems and mitochondria, finally leading to neurodegenerations. Due to neurodegenerations, HD patients suffer clinical symptoms, including motor and cognitive deficits

Full length of HTT/mHTT contains several protease cleavage sites, where capsase-3, caspase-6, calpain or aspartic proteases could digest full length HTT to form different sizes of truncated N-terminal HTT/mHTT fragments [3, 12–14]. In HD, over-expanded polyQ accelerates N-terminal mHTT fragments to transform to β-sheet confirmations from α-helical coiled coils, and easily leads to protein misfolding. Especially, the first 17 residues of N-terminal HTT preferentially adopt the β-sheet conformations as the polyQ increases, further enhancing the accumulation of protein misfolding [15]. These misfolded mHTTs generate dimers, trimers or oligomers, and form pathological aggregates. As a result, the misfolded mHTTs disrupt gene regulation, vehicle trafficking, synaptic plasticity, protein clearance, neurogenesis, etc., and finally lead to cellular dysfunctions and death [3, 16–19] (Fig. 1). Since this protein misfolding is a critical cause for HD, several therapeutical strategies have addressed on this aspect.

#### Mitochondrial dysfunctions

Mitochondrial dysfunction is a common characteristic in different neurodegenerative diseases, such as Alzheimer's disease (AD), Parkinson’s disease (PD) and Spinocerebellar Ataxia [20–22], and failure of mitochondrial functions results in increases of oxidative stress and deficits of energy production, further leading to neurodegenerations. In different HD models, several reports have shown the increase of oxidative stress and deficits of mitochondria in this disease [23–25]. These deficits include the impairments on mitochondrial biogenesis, dynamics, membrane potential, electron transport chains, adenosine triphosphate (ATP) productions, etc., and finally affect cellular functions, neuropathogenesis and clinic symptoms [24–27]. Due to the critical roles of mitochondrial functions in HD pathogenesis, several treatments targeting on this aspect have been demonstrated. For example, activation of mitochondria-related genes, such as Sirtuin 3 (SIRT3), Peroxisome proliferator-activated receptor gamma co-activator-1 alpha (PGC1alpha), Uncoupling protein (UCP), has shown to alleviate HD symptoms in different models [23, 24, 28], highly suggesting improvement of mitochondrial functions is an important therapeutical strategy for HD (Fig. 1).

#### Abnormal protein degradation

Protein degradation is an important step to control protein quality, and further maintains the cellular functions of proteins in different tissues. Ubiquitin-proteasome system (UPS) and autophagy, also known as macroautophagy, are two important systems responsible for protein degradations inside cells. The UPS clears up short-lived proteins by tagging a ubiquitin activating enzyme (E1), a conjugating enzyme (E2) and a ligase (E3), and further degrades these proteins in 26S proteasomes in the nucleus or cytoplasm. The autophagy system forms autophagosomes and autolysosomes, and then degrades long-lived proteins in lysosomes of cytoplasm. Based on previous studies, several different neurodegenerative diseases show poor treatments due to loss of functions in these two systems [29, 30]. In HD, mHTT has been reported to be degraded slowly, and impairment of two protein degradation systems has been found in different HD models [17, 31–34]. For example, a deficiency of a unique α-amine E2 enzyme, UBE2W, is highly correlated with the accumulation of mHTT [35], and polyQ of mHTT has been shown to block the activity of proteasomes [36], indicating the impairment of the UPS system in HD. In addition, mHTT disrupts autophagosome dynamics and vesicle trafficking, and also decreases the initiation of autophagy [37–39], indicating the impairment of the autophagy system. As a result, enhancement of these two protein degradation systems is also considered as a potential strategy of therapy in HD (Fig. 1).

### Current and potential treatments

#### Current treatment/drugs

Till now, there is no treatment to cure HD, and the clinic treatments only could alleviate symptoms to improve the quality of life (Table 1). Chorea is one of motor symptoms in HD, and decreased neurotransmission of dopamine has shown the reduction of chorea [40], indicating decrease of dopamine signaling is a therapeutic target for HD patients. Tetrabenazine (TBZ) and Deutetrabenazine, both approved by the Food and Drug Administration (FDA), are inhibitors of the vesicular monoamine transporter type 2 (VMAT2), and functions through depletion of dopamine in the presynaptic terminals to improve chorea [40, 41]. In addition, dopamine receptor blockers, such as tiapride, haloperidol, fluphenazine, olanzapine and risperidone, are also used to treat HD to suppress chorea. Since dopamine receptor blockers belong to one type of antipsychotics, these drugs also alleviate comorbid psychiatric symptoms in HD [40, 42]. Besides to the decrease of dopamine signaling, suppression of glutamate transmission is also a strategy to reduce chorea. These drugs used in clinic include riluzole and amantadine [8, 43]. Moreover, alleviations of psychiatric and cognitive symptoms are also important to improve quality of life. For example, selective serotonin reuptake inhibitors (SSRIs) or serotonin-norepinephrine reuptake inhibitors (SNRIs) has been used to deal with depression and anxiety, and above dopamine receptor blockers have used as antipsychotics in HD [40, 43]. Since these drugs target on alleviation of symptoms only, it is important to develop treatments to cure HD.

**Table 1:** Current and potential treatments for HD

Current and potential treatments for HD		
Current drugs	Working mechanisms	References
Tetrabenazine; Deutetrabenazine	VMAT2 inhibitors	[41, 5]
Tiapride; Haloperidol; Fluphenazine; Olanzapine; Risperidone	Dopamine receptor blockers	[42, 40]
Riluzole; Amantadine	Suppression of glutamate transmission	[8, 43]
Cell therapy	Cell replacement; Secretion of beneficial factors	[44, 44–46]
Gene therapy	Reduction of mutant HTT; Enhancement of cellular functions	[47, 47–55]

#### Cell therapy

Cell-based therapy, which may provide structural and functional reconstruction in brain regions, is a potential strategy for neurodegenerative diseases (Table 1) [20, 44, 45]. In HD, these related studies have been examined in different animal models. For example, intranasal administration of mesenchymal stem cells or intrastriatal administration of dental pulp stem cells has ameliorated neuropathological and behavioral phenotypes in different HD rodent models [46], and these results highly suggest potential applications in clinic. Indeed, several worldwide clinical trials of cell transplantations in HD have been conducted, and showed the amelioration of brain structures, neural circuitry and motor functions [45]. Although some HD patients showed long-lasting improvements of their symptoms, graft failure was also observed in several patients due to different individual characteristics, immune responses, graft protocols or cell sources in different trials [45]. As a result, the guide line for cell-based therapy is still being developed.

#### Gene therapy

Since the HD is a monogenetic disease, mHTT-lowering strategy is considered as a potential direction for the therapy. Taking advantages of different molecular techniques, such as antisense oligonucleotides, gene deletion and RNA interference, these alterations suppress the expression of mHTT and rescue HD phenotypes in different models as well (Table 1) [47–50]. Based on the information via ClinicalTrials.gov provided by the U.S. National Library of Medicine, antisense oligonucleotides designed for mHTT are already in phase II and III of clinical trials (NCT02519036, NCT03225833, NCT03225846, etc), highly suggesting foreseeable potential of these therapies. In addition to targeting on mHTT, increases of neuroprotective effects through other functional genes, such as neurotrophic factors, growth factors, anti-oxidative stress genes and microRNAs, have also alleviated HD symptoms [25, 51–55]. These results suggest gene therapies may not only offer temporary relief, but also cure the root cause in HD.

## MicroRNA

### Cellular biofunctions

#### microRNA biogenesis

microRNA (miRNA) is a small non-coding RNA, and plays an important role in gene regulation. miRNA transcripts are generated from non-coding regions of primary miRNAs (Pri-miRNAs), and the Pri-miRNAs are processed by a nuclear ribonuclease III enzyme, Drosha, to form hairpin precursor miRNAs (Pre-miRNAs) inside nucleus. Pre-miRNAs are transported to cytoplasm by exportin proteins, such as exportin 5, and then are processed again by the Dicer to form mature miRNAs. Mature miRNAs further interact with a RNA-induced silencing complex (RISC), and bind to target mRNAs to mostly cleave mRNA or suppress translation at the post-transcriptional level [56]. Although it is well known miRNAs function through downregulating gene expression, miRNAs also have been reported to offer opposite effects to upregulate translation during different stages of cell cycle [57]. Notably, one miRNA usually has targeted on multiple genes (Winter, Jung et al. 2009), suggesting the importance to control the expressions of miRNAs under physiological conditions.

#### microRNAs related to mitochondrial functions

**Fig. 2:**
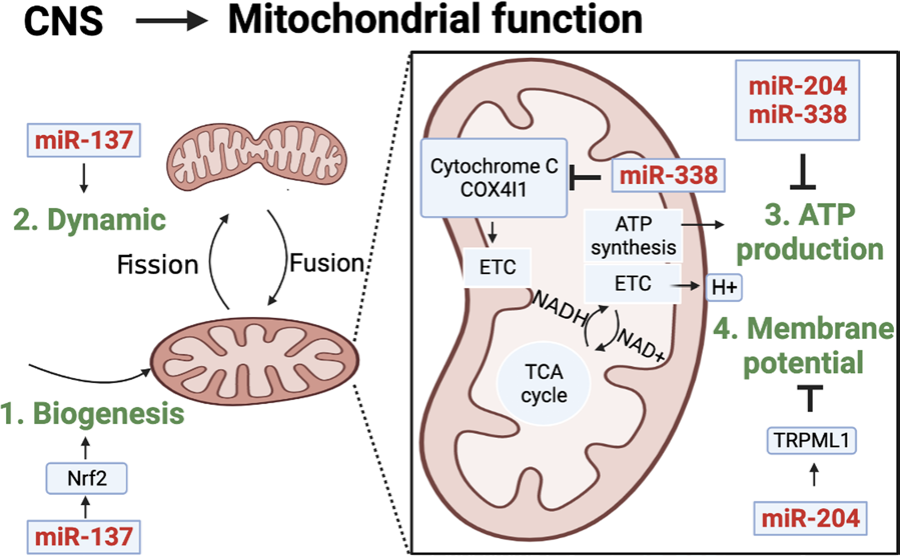
microRNAs regulate mitochondrial functions in CNS. Different miRNAs involve in mitochondrial functions, such as mitochondrial dynamic, biogenesis, ATP production and membrane potential

As described above, mitochondrial functions could be determined through mitochondrial biogenesis, dynamics, membrane potential and ATP productions. These mitochondrial functions are closely regulated by different miRNAs during neuronal development and disease progression. For example, miR-137 upregulates nuclear factor erythroid 2-related factor 2 (NRF2) to enhance mitochondrial biogenesis, and also further modulates mitochondrial fusion and fission to affect mitochondrial dynamics in neural stem cells [58]. In addition, miR-338 targets to cytochrome-c oxidase subunit 4I1 (COX4I1) to regulate mitochondrial ATP production in brains [59]. Furthermore, miR-204 is upregulated to target transient receptor potential mucolipin-1 (TRPML1) to damage mitochondrial membrane potential and ATP production in AD, and suppression of miR-204 rescues those mitochondrial damages and reactive oxygen species (ROS) productions through upregulation of TRPML1 [60]. These results indicate brain-enriched miRNAs, such as miR-137, miR-338 and miR-204, play critical roles to maintain mitochondrial biogenesis and biofunctions during brain development, and unbalance of these miRNA regulations leads to the progression of neuronal diseases (Fig. 2).

#### microRNAs related to protein degradations

**Fig. 3:**
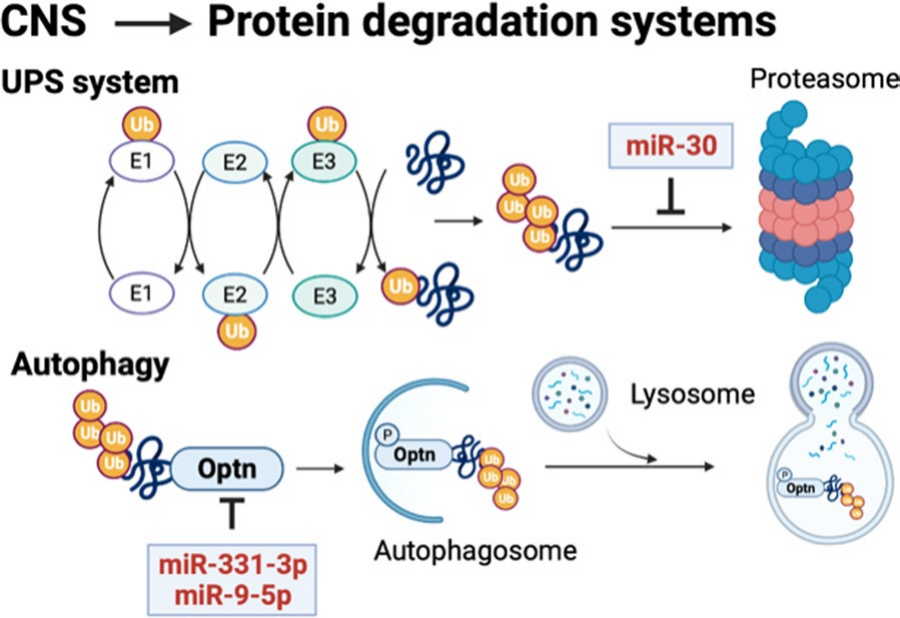
microRNAs regulate two protein degradation systems in CNS. Different miRNAs involve at different stages of different protein degradation systems, such as the UPS and autophagy systems

Two protein degradation systems, UPS and autophagy, are regulated by several miRNAs as well. Based on previous reports, several miRNAs have shown to target different E1, E2 or E3 enzymes of UPS to affect different neuronal functions [61–63]. Similar to UPS, the autophagy system is also closely regulated by miRNAs in the neuronal system, and autophagy-related proteins, such as Sequestosome 1 (Sqstm1), Optineurin (Optn), BACE1 and ATG5, are directly controlled by miRNAs to affect autophagic activity [64, 65]. Due to dysfunctions of these protein-degradation-related miRNAs, the cells, including neurons, glia cells and microglia cells, in CNS fail to process genesis, migration, maturation or advanced interactive functions. For example, higher expression level of miR-30a-5p has shown to worsen UPS to downregulate glutamate transporter 1 (GLT-1), further leading to glutamate excitotoxicity [62]. In addition, higher expression levels of miR-331-3p and miR-9-5p have shown to target autophagy regulators, Sqstm1 and Optn, further impairing autophagic degradation activity [64]. These results all support the critical roles of miRNAs in the cellular functions of protein degradations (Fig. 3), implying a potential application to adjust these cellular functions through miRNAs to treat different neuronal diseases.

### microRNA toward neuronal diseases

#### microRNAs in neurodegenerative diseases

Abnormal expression of global miRNAs or specific miRNAs has been reported to cause neurodegenerative diseases. Taking advantages of gene-modified animals, deletion of Dicer, which causes abnormal expression of global miRNAs, has led to neuronal degenerations, impairments and symptoms [66–68]. Deletion of specific miRNAs, such as miR-124-1 or miR-8, has also shown to develop neuronal degenerations and apoptosis in different models [69, 70]. These indicate loss of miRNA functions could be a potential disease-causing factor for neurodegenerative diseases; however, these is still limited information regarding to mutations of specific miRNAs lead to neurodegenerative diseases in clinic. This provides an insight that searching mutations in non-coding miRNAs could be one of potential directions for identification of disease-causing factors in the sporadic type of neurodegenerative diseases. Moreover, although miRNAs might not be main causes for neurodegenerative diseases, these diseases, including AD, PD and HD, do lead to alterations of miRNA expression profiling, which further regulates downstream cellular functions and finally leads to disease-specific pathology and symptoms [61, 62, 64, 71, 72]. Since the expression profiling of certain specific miRNAs is associated with the disease progression, neuropathology or symptoms, these miRNAs could be used as biomarkers for disease diagnoses and also could be applied for therapy potentially.

#### microRNAs in Huntington’s disease

The expression profiling of miRNAs in HD patients has been reported in several studies, and altered miRNAs are highly associated with the regulation of molecular or pathological phenotypes [73–75]. In addition, global miRNA alterations have also been reported in HD because of the accumulation of Argonaute-2 (AGO2) protein, which is a critical component for RISC [76]. These results show HD could lead to abnormal expression of global miRNAs or specific miRNAs as well. As a result, miRNAs are recently addressed as biomarkers to indicate the progression of HD in clinic [73, 77].

Due to limited manipulations of miRNA alterations in HD patients, miRNA studies have been widely demonstrated in different animal models. In transgenic monkey studies, HD transgenic monkeys showed downregulation of miR-128a and upregulation of miR-196a, which is similar in HD patients [61, 78, 79]. In transgenic mouse studies, miR-9/9*, miR-124 and miR-132 have been shown to be suppressed in both human HD patients and mouse models [75, 79, 80]. Since HD patients and animals display certain similar miRNA profiling, these animal models have been used to investigate that miRNAs as potential therapeutical targets in HD. Indeed, the treatments of miR-132 in R6/2 HD transgenic mice show improvements of behavioral symptoms and delay of disease progression [81]. In addition, miR-196a also improves molecular, neuropathological and behavioral phenotypes in HD transgenic mice through enhancements of neuronal cytoskeletons [52, 61]. Most interestingly, several studies have addressed on artificial miRNAs targeting on human HTT [50, 82–84]. Although these miRNAs do not selectively target on mutant HTT and do reduce the expression of normal HTT, these miRNAs offer functional improvement, such as decrease of aggregates and alleviation of HD symptoms, and also do not lead to severe side-effects. Indeed, this strategy, as known as AMT-130, of non-selectively lowering HTT using artificial miRNAs has been undergone in a clinical phase 1/2 trial (NCT04120493) in 2021[85], and aims to examine the safety and efficacy of this miRNA therapy in early stage of HD patients. Due to the similar approach examined in different animal models already, physicians are looking forward to seeing positive results. Overall, since these *in vivo* data show strong evidence to alleviate HD progression in animal models, it highly raises this potential therapy using miRNA-based strategies in HD.

### Potential treatments using miRNAs

#### Different types of miRNA mimics or inhibitors

miRNAs have been considered as a pharmacologic strategy against diseases, and different types of miRNA mimics or inhibitors altering the expression of miRNAs have been developed. Regarding to miRNA mimics, mature form of miRNAs is commonly synthesized to deliver into cells or tissues. However, the half-life of these raw mimics is usually less than 30 min due to degradation by RNases, resulting in limited effects of functional miRNAs [86]. As a result, modified miRNAs have been developed to prevent RNase attacks. For example, phosphorothioate (PS) RNA is generated through using sulfur in the phosphodiester bonds of the RNAs in order to decrease the attacks of RNases, and modifications in 2’-OH group of RNAs to form 2'-O-methyl (2'-O-Me) RNAs are also used to increase the miRNA stability, further enhancing the effects of miRNA mimics [86–88]. Regarding to miRNA inhibitors, antisense RNAs are broadly used to block miRNAs; however, quick degradation of antisense RNAs is observed as well, which is similar to that of miRNA mimics. Therefore, PS and 2’-OMe RNA techniques are also broadly used as miRNA antisense inhibitors to block the functions of miRNAs [86, 88]. Even more, new modified mesyl phosphoramidate RNA targeting to miRNAs has shown more efficient to block miRNA functions [89]. These results suggest the increase of miRNA stability to prolong half-life is a critical step to apply miRNA therapy, and synthetic biology would also play an important role for this application.

#### Delivery systems

Brains with blood–brain barrier (BBB) result in obstacles for drug delivery into cells; as a result, a suitable delivery system is necessary to transport miRNAs into target brain cells. Nanoparticle is a powerful platform able to penetrate BBB to deliver RNA-based treatments into brain regions, and has been developed for more than 10 years. Several types of nanoparticles carrying miRNAs have been examined in different models of neuronal diseases *in vivo*, including quercetin-conjugated superparamagnetic iron oxide nanoparticles in AD [90], traceable polymeric nanoparticles in PD [91], poly(lactic-co-glycolic acid) nanoparticles in stroke [92], etc. These materials could not only show high capacity and low cytotoxicity to deliver miRNAs into brain regions, but also harbor other biological purposes, such as antioxidant effects and distribution monitoring, showing that nanoparticles with dual or more functions are a trend to develop this type of delivery systems.

In addition to nanoparticles, delivering miRNAs by viral systems has also been applied in several neuronal diseases. Especially, adeno-associated viruses (AAVs) are most often used because higher titer of AAVs is relatively easier generated, higher levels of miRNAs could be produced in AAVs, AAVs do not easily integrate into genomes and AAVs are relatively safer than other viral systems. Therefore, AAVs, such as Luxturna, has been approved by FDA to apply in clinical trials [93]. In animal studies, AAVs expressing miRNAs have been broadly demonstrated to target neuronal diseases. For example, AAVs carrying miR-23a have improved neuropathology and survival rate in spinal muscular atrophy (SMA) mice *in vivo* [94]. AAV-miRNAs targeting on spinocerebellar ataxia type 3, another polyglutamine disease, have shown to reduce the expression of disease-causing gene in different models [95]. In HD, AAV-miRNAs against mutant HTT have also been examined in different *in vivo* models, and shown different beneficial effects on molecular or neuropathological phenotypes [96–98]. Due to approval of clinical trials previously, these animal studies highly bridge the potential applications of AAVs carrying miRNAs to target neuronal diseases in clinic.

Exosomes, which are small extracellular vesicles with a diameter of less than 100 nm, are an emerging carrier to deliver miRNAs into brains recently because of lipid bilayer structures. Exosomes carry proteins, mRNAs, miRNAs, etc., and deliver these materials to specific target cells to further control cellular signals or gene regulations [86, 99]. Since exosomes are endogenous components inside cells, higher stability, lower immunogenicity and lower cytotoxicity are advantages to apply to deliver miRNAs in vivo. As a result, the exosome-based delivery systems carrying miRNAs have been used to protect brains from injury in several neuronal diseases, such as brain glioblastoma, ischemia or hemorrhage [100–103]. In HD mouse models, this exosome system has been applied to deliver miR-124 to suppress the target gene, RE1-silencing transcription factor (REST) which represses neuronal genes, in R6/2 HD transgenic mice; however, this treatment does not improve HD behaviors [104]. Due to rapid progression of exosome-related studies, optimization of this strategy is anticipated to apply to deliver miRNAs efficiently and safely in vivo.

#### Limitations and challenges

**Fig. 4:**
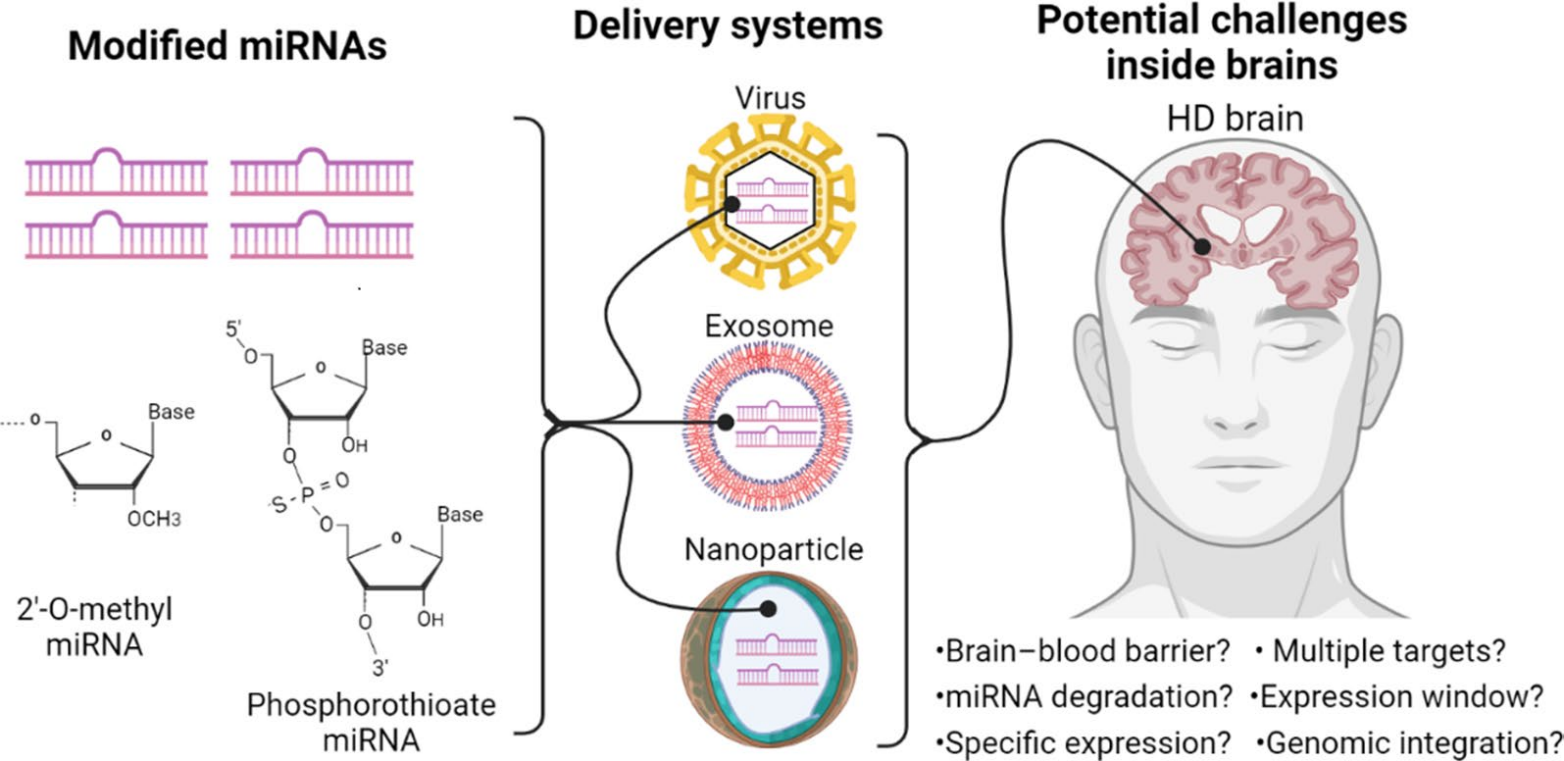
miRNA-based therapy may encounter potential challanges in HD. miRNA-based therapy needs to overcome the miRNA instability, suitable delivery systems and functional effects inside brains. Nucleotide modifications, such as 2'-O-methyl- and phosphorothioate- structure, may increase the stability of miRNAs. Delivery systems, such as viruses, exosomes and nanoparticles, may faciliate the efficiency and safety to transport miRNAs into brains. Most importantly, several potential challenges which may be encountered inside brains are listed

Although miRNAs show several advantages for potential applications of therapy in neuronal diseases, there are still limitations and challenges (Fig. 4). The major limitation of miRNA applications is one miRNA usually has multiple targets. As a result, treatments of miRNAs may encounter unexpected side-effects due to multiple bindings in different direct targets. Therefore, safety or toxicity studies should be evaluated before clinical trials. Indeed, certain miRNA treatments for HD have been examined. For example, the side-effects of miRNAs targeting on HTT have been demonstrated in mice, rats, minipigs and non-human primates, and results show the tolerability in these in vivo models [50, 83, 84]. However, this characteristic may oppositely be considered as an advantage because miRNAs might also target multiple pathways to regulate downstream phenotypes. For example, miR-196a has bound to different target genes to decrease aggregates of mutant HTT, improve mitochondrial dynamic, enhance neurite outgrowth, etc., to alleviate the progression of HD in different models [52, 61, 105, 106]. This indicates miRNAs may function through different regulatory pathways to further provide synergistic effects.

In addition, the miRNAs are relatively unstable, and the half-life of miRNAs is shorter as described in “Different types of miRNA mimics or inhibitors”. Although modified miRNAs have efficiently extended the half-life, long-term expression of these miRNAs is desired if miRNAs are used as a therapeutical strategy to cure genetic diseases. The viral system, such as AAV- or lentivirus-based system, is one of options to achieve long-term expression. The AAV delivery system is able to continuously express exogenous transgenes for more than one year without integrating into host genomes [107], and the lentivirus-based system even constitutively expresses transgenes due to integrating into genomes [108]. However, the random integration of lentiviruses into genomes may disrupt the endogenous gene expressions [109], which raises another drawback of the viral system.

Moreover, cell-, tissue- or region-specific expression of miRNAs is also a challenge for treatments of miRNAs in CNS. Although miRNAs could be stereotaxically injected into regions of interest, the invasive penetrations are not always allowed in CNS. To overcome this obstacle, cell- or tissue-specific promoters could be manipulated to achieve this purpose if the viral delivery systems are used to transport miRNAs. Besides, targeted exosomes, which could transport siRNAs to specific tissues via targeting peptides, are an option to reach this goal as well [110]. Additionally, specific coating of antibodies, ligands, etc., on nanoparticles to carry miRNAs is also a potential strategy to deliver to specific cells/regions [111, 112]. However, these platforms are still needed to be optimized to increase specificity, efficacy and safety as applied in clinic.

## Conclusion

HD is a devastating neurodegenerative disease, and leads to unimaginable burdens to patients, families and societies. Since HD displays several pathological phenotypes, such as protein misfolding, mitochondrial dysfunctions and abnormal protein degradation, it raises a potential therapy for HD if a treatment could target multiple pathological phenotypes simultaneously. miRNAs have multiple target genes, and may function through several pathways to influence different phenotypes simultaneously. With the development of modified miRNAs and delivery systems, miRNAs raise a potential direction for therapy to treat HD; however, this idea is still at the proof-of-concept stage. In future, the optimization of miRNA structures and delivery systems would be a critical step to apply to the clinic. In addition, overcoming drawbacks and limitations would also be an important step to apply miRNA therapy.

## Data Availability

Not applicable.

## Abbreviations

HD: Huntington’s disease
miRNA: MicroRNA
HTT: Huntingtin
*mHTT*: Mutant *HTT*
ploy Q: Polyglutamine
CNS: Central nervous system
AD: Alzheimer's disease
PD: Parkinson’s disease
ATP: Adenosine triphosphate
SIRT3: Sirtuin 3
PGC1alpha: Peroxisome proliferator-activated receptor gamma co-activator-1 alpha
UCP: Uncoupling protein
UPS: Ubiquitin–proteasome system
TBZ: Tetrabenazine
FDA: Food and Drug Administration
VMAT2: Vesicular monoamine transporter type 2
SSRIs: Selective serotonin reuptake inhibitors
SNRIs: Serotonin-norepinephrine reuptake inhibitors
Pri-miRNAs: Primary miRNAs
Pre-miRNAs: Precursor miRNAs
RISC: RNA-induced silencing complex
NRF2: Nuclear factor erythroid 2-related factor 2
COX4I1: Cytochrome-c oxidase subunit 4I1
TRPML1: Transient receptor potential mucolipin-1
ROS: Reactive oxygen species
Sqstm1: Sequestosome 1
Optn: Optineurin
GLT-1: Glutamate transporter 1
AGO2: Argonaute-2
PS: Phosphorothioate
2'-OMe: 2'-O-methyl
BBB: Blood–brain barrier
AAV: Adeno-associated virus
SMA: Spinal muscular atrophy
REST: RE1-silencing transcription factor
